# Tumor-Infiltrating Lymphocytes and Neutrophils and Their Location in Canine Mammary Neoplasms with a Solid Arrangement: A Prognostic Factor?

**DOI:** 10.3390/ani15020287

**Published:** 2025-01-20

**Authors:** Mayra C. Flecher, Marina P. Reys, Débora Balabram, Karen Y. R. Nakagaki, Geovanni D. Cassali

**Affiliations:** 1 Department of General Pathology, Laboratory of Comparative Pathology, Federal University of Minas Gerais, Belo Horizonte 31270-091, Minas Gerais, Brazil; mayraflecher@gmail.com (M.C.F.); marinareys13@gmail.com (M.P.R.); karenyumi@ymail.com (K.Y.R.N.); 2 Department of Veterinary Medicine, Universidade Vila Velha, Vila Velha 29102-920, Espírito Santo, Brazil; 3 Department of Surgery, School of Medicine, Federal University of Minas Gerais, Belo Horizonte 31270-091, Minas Gerais, Brazil

**Keywords:** mammary neoplasms, tumor-infiltrating lymphocytes, survival analysis, CD3, CD79, neutrophils, metastasis, dogs

## Abstract

In canine mammary neoplasms, greater inflammation is associated with higher histological grade, lymphatic invasion, and metastases. This retrospective study assessed the density of peri- and intratumoral tumor-infiltrating lymphocytes (TILs), tumor-associated neutrophils (TANs), and CD3^+^ and CD79^+^ lymphocytes in canine mammary neoplasms with a solid arrangement, and associated such data with histological types, immunophenotype, prognostic factors, cyclooxygenase-2 (Cox-2) expression and overall and cancer-specific survival. Sixty-one neoplasms with a solid arrangement were classified as malignant myoepitheliomas (6/9.8%), solid papillary carcinomas (8/13.1%), carcinomas with a solid pattern (9/14.8%), basaloid carcinomas (BC) (19/31.1%), and malignant adenomyoepitheliomas (19/31.1%). Intra- and peritumoral TILs, TANs, and TCD3^+^ and BCD79^+^ lymphocytes were counted, and based on the resulting median, the neoplasms were divided into low or high cell infiltration. BCs had the lowest density of intratumoral TILs (*p* = 0.02), and luminal B neoplasms showed a significantly higher density of intratumoral TCD3^+^ than luminal A cases. Neoplasms with a higher density of peritumoral CD3^+^ and CD79^+^ had significantly greater proliferative activity. High infiltration of intratumoral BCD79^+^ lymphocytes was related to nodal metastasis (*p* = 0.03). Intratumoral TILs and TCD3^+^ were associated with shorter survival time. Therefore, intratumoral lymphocyte infiltration is possibly an important feature in the progression of cancer and influences the survival in bitches with solid arrangement neoplasms.

## Simple Summary

Mammary neoplasms are the most common tumors in female dogs, and mammary neoplasms with a solid arrangement encompass histological types with high proliferative activity that commonly lead to metastasis. In human breast tumors, in addition to the histological type, the characterization of the inflammatory infiltrate and its location are considered prognostic and predictive factors. In veterinary medicine, little is still known about the influence of inflammation on cancer progression and its relationship with histological types. In the present study, we observed that neoplasms with a solid arrangement presenting with a high density of lymphocytes and neutrophils, especially in the intratumoral area, are associated with shorter survival time, and that the density of intratumoral B lymphocytes is proportional to the presence of lymph node metastasis. Therefore, tumor inflammation should be considered as a factor influencing the progression of mammary neoplasms in female dogs.

## 1. Introduction

The characterization of tumor microenvironment through the evaluation of infiltrating leukocytes is already established for some types of neoplasms in the human species, such as colorectal carcinomas [1,2,3], oral and cutaneous squamous cell tumors [4,5], renal, head and neck, hepatocellular, pancreatic tumors [6], and mammary [7,8] and urothelial bladder cell tumors [9].

The presence of tumor-infiltrating lymphocytes (TILs) and their subtypes is associated with morphological (histological grade, size, pleomorphism, and mitotic count) and immunophenotypic characteristics of breast tumors. Thus, TILs are considered prognostic and predictive factors in women’s breast cancers [10,11,12,13]. In women, TILs tend to be present in a greater proportion in neoplasms without the expression of hormone receptors and show an overexpression of HER-2 and a higher percentage of Ki-67 [14,15,16]. Also, greater inflammation in triple-negative neoplasms (TN) and tumors with HER-2 overexpression, especially in the intratumoral area, is associated with a better response to chemotherapy treatment [10,13,14]. In female dogs, the highest intensity of TILs is observed in higher histologic grade neoplasms, with lymphatic invasion [16,17] and shorter survival time, a characteristic that is considered an independent prognostic factor. In addition, the highest percentage of B cells (CD79^+^) was seen in carcinoma in mixed tumors with metastasis to lymph nodes when compared to carcinoma in mixed tumors without metastasis to lymph nodes [18]. However, little is known about the influence of inflammation and the relationship of inflammation and immunophenotypes in mammary neoplasms in female dogs. No relationship was found between TIL and molecular subtypes in one study [16], but in another, the peritumoral inflammation was shown as an independent prognostic factor for bitches with triple-negative tumors [19].

Few studies approach tumor-associated neutrophils (TANs) in women breast cancer, and most of those studies are conducted in vitro and in animal models. Similarly to lymphocytes, neutrophils may be associated with a longer or shorter survival time, depending on the type of cytokine or receptors present in the microenvironment, [20,21]. Estrogen receptor-positive neoplasms, in advanced stages, show an increase in the amount of TGF-ß in the microenvironment, favoring the chemotaxis and polarization of N2 neutrophils, which favor neoplastic dissemination and pre-metastatic niche formation [22]. In the few studies involving women, the increase in the average amount of neutrophils is associated with TN- and HER-2-positive tumors [23], and a high density of TANs is associated with shorter disease-free time [24]. In bitches, the amount of neutrophils was higher in carcinoma in mixed tumors and tubular carcinomas compared to solid carcinomas and benign mixed tumors [25].

Although the quantification of TILs in H&E slides is an important parameter and is associated with prognostic and predictive factors in breast cancer in women, it is not enough to identify lymphocyte subtypes, which are fundamental for understanding the immune response to cancer. In the present study, we quantified tumor-infiltrating lymphocytes (TILs) and tumor-associated neutrophils (TANs) in the peri- and intratumoral areas, on H&E slides, and on slides with immunohistochemistry for CD3^+^ and CD79^+^ lymphocytes. Afterwards, we dichotomized high- and low-density inflammations to associate them with the histological types of neoplasms with a solid arrangement, immunophenotypes, prognostic factors (tumor size, regional lymph node metastasis, Ki-67 index, and mitotic count), Cox-2 immunostaining, and overall and specific survival time for bitches.

## 2. Material and Methods

### 2.1. Selection and Classification of Samples and Clinical Follow-Up

The criteria for sample selection, classification of neoplasms, and clinical follow-up of the animals were based on previous study, which included 65 samples [26]. However, four samples were entirely used up in the first part of the research, and in the present article, 61 of these samples were used. The sixty-one canine mammary neoplasms with a solid arrangement, diagnosed between 2011 and 2022, were selected from the collections of the Comparative Pathology Laboratory at the Institute of Biological Sciences of the Federal University of Minas Gerais (UFMG), the Pathology Laboratory of Vila Velha University (UVV), the Celulavet Laboratory, and the Center for Research in Mammary Oncology at the Federal University of Bahia (UFBA). Only samples from female dogs previously diagnosed with neoplasms classified as solid carcinoma [27] or basaloid carcinoma [28], for which presented macroscopic description information of location and size of neoplasms was available and evaluation of regional lymph nodes was performed, were included. Female dogs presenting with other histological types of more aggressive mammary-origin neoplasms associated with shorter survival times [28,29], non-mammary malignant neoplasms, and those that had been treated prior to surgery were excluded from the study.

From these criteria, histological slides stained by hematoxylin and eosin (H&E) were re-evaluated by two veterinary pathologists (GDC—board-certified by the Brazilian Association of Veterinary Pathology, MCF—residency in Veterinary Pathology and Ph.D. in Pathology) and reclassified into carcinomas with a solid pattern (CSP), basaloid carcinomas (BC), solid papillary carcinomas (SPC), malignant adenomyoepitheliomas (MAME), and malignant myoepitheliomas (MME) based on morphological and phenotypic patterns (Figure S1) [30]. Neuroendocrine carcinomas were not evaluated in the present study due to the small number of samples. MAME may present benign epithelial areas, making it difficult to assess its epithelial invasion area, and MM is composed of more than 90% myoepithelial tissue [30]. Therefore, as these histological types do not fit the standardization of histological grading used for invasive carcinomas [31], grading was not evaluated in this study. In all 61 cases, the regional lymph nodes were also evaluated for the detection of metastasis. Information on neoplasm size was provided by the laboratory where the samples came from, and tumor size was stratified into T1 (< 3 cm), T2 (between 3 and 5 cm), and T3 (> 5 cm), following the TNM system [32]. For neoplasms larger than T2, more than one histological slide was evaluated; the slide containing the best sample of approximately 2.0 cm with less necrosis and fixation and crushing artifacts was selected. As utilized in the grading system by Elston and Ellis, mitotic counting was performed in 10 fields (2.37 mm^2^) at the tumor periphery in continuous fields. As information on distant metastasis assessment was incomplete, the complete staging was not performed in the present study.

The time in days from the date of surgery until the death of the dog from any cause and from neoplasia-related cause was calculated for overall survival and specific survival, respectively [17,18,19]. Cause of death related to neoplasia means spontaneous death or euthanasia due to local progression or systemic deterioration in animals with metastatic disease. The follow-up was carried out for a period of at least one year. Information was obtained from the responsible tutor and/or the veterinary clinician.

### 2.2. Immunohistochemistry

Four-micrometer-thick sections were obtained from the paraffin blocks and placed on gelatinized slides, submitted to deparaffinization, dehydration in alcohol and xylol, and manual staining for the markers following previously validated protocols [26,30,33]. The antigenic recovery was performed in moist heat, within citrate pH 6.0. Novolink^®^ kit solution (Leica) was used as the secondary antibody and detection system. Evaluations were performed under an Olympus BX-40 microscope coupled with a Spot Insight Color digital camera and with the aid of SPOT capture software version 3.4.5.

In order to establish the immunophenotype of the neoplasms, immunohistochemistry was performed for estrogen (ER, ready-to-use, clone Ep1, Dako^®^) and progesterone (PR, 1:50, clone hpra2, Thermo Fisher^®^) receptors, cell proliferation factor Ki-67 (1:50, clone mib 1, Dako^®^), and epidermal growth factor 2 (HER-2, 1:200, clone c erb 2, Thermo Fisher^®^) in all selected samples. Immunolabeling for cyclooxygenase 2 (Cox-2, ready-to-use, clone Sp21, Invitrogen^®^) was also performed on 59 samples. The antibodies are well standardized for the technique used in the laboratory routine, and the antigenic specificity has already been tested, as shown in previous publications [26,30,33]. Due to wear of the paraffin block during the study, two cases lacked enough material for this analysis. For the evaluation of ER and PR labeling, a scoring system considering the number of cells with positive nuclear labeling (negative: <1%, +: 1–25% of labeled cells; ++: 26–50% of labeled cells, + ++: 51–75%; + +++: >75% of labeled cells) was adopted (Figures S2 and S3) [34]. Ki-67 was determined by the percentage of cells with nuclear staining in a count of 500 cells through photomicrographs made in a 40× (0.096 mm^2^) objective hot spot analyzed with Image J^®^ imaging system, and the cutoff point ≥20% of labeled cells was used for classification as high and low proliferation (Figure S4) [35]. HER-2 expression followed the guidelines of membrane marking score, considering scores 0, 1+, and 2+ as negative and 3+ as positive (Figure S5) [36]. Then, the neoplasms were categorized into luminal A (ER and/or PR positive, HER2 negative, and Ki-67 <20%); luminal B HER2 negative (ER and/or PR positive, HER2 negative, and Ki-67 ≥20%), luminal B HER2 positive (ER and/or PR positive, HER2 positive, and any Ki-67), HER2 overexpressed (ER and PR negative, and HER2 positive), and triple negative (TN) (ER, PR, and HER2 negative) [26,35]. Normal mammary gland was used as positive control, while a buffer solution was used as negative control instead of the primary antibody. The Cox-2 assessment was based on a semi-quantitative evaluation of labeled tumor cells in the cytoplasm. Percentage of labeled tumor cells throughout the evaluated fragment (0= absent, 1= less than 10% of cells labeled, 2= 10 to 30% of cells labeled, 3= 31 to 60% of cells labeled, 4= more than 61% of cells labeled) and intensity score (0= absent, 1= weak (+) labeling, 2= moderate (++) labeling, and 3= strong (+ ++) labeling) were defined. The final score is established by multiplying the distribution by intensity (Figure S6) [37].

Anti-CD3 (1:200, Dako^®^, A052) antibodies were used for the identification of T cells, while anti-CD79 antibodies were used (1:400, Biolegend^®^, HM47) for the identification of B cells. Normal dog lymph nodes were used as positive and negative controls. A buffer solution was used for negative control instead of the primary antibody. The negative control was prepared following the same technique described in the determination of immunophenotypes.

### 2.3. Characterization of the Infiltrate in Histological Slides and Immunohistochemistry

Images of eight hot spots at 600× (60× objective and 10× ocular) were captured from the H&E slides and contained a fragment of each neoplasm for quantification of TILs and TANs in the peri- and intratumoral areas, separately. The 600× magnification (0.087 mm^2^) provides better definition of the cell types. Hot spot stands for the field of the fragment that presents a higher concentration of inflammatory cells. For the quantification of T and B cells labeled for CD3^+^ and CD79^+^, images of eight hot spots were captured at 400× (0.196 mm^2^), following the same capture methodology made on the H&E slides. Photomicrographs were taken by a Spot Insight Color digital camera adapted on the Olympus BX-40 microscope with the aid of SPOT capture software version 3.4.5 [18].

The identification of inflammatory cells (neutrophils and lymphocytes) on the histological slides was based on the morphological aspect of each cell. In the slides submitted to immunolabeling, lymphocytes were positive when the label was identified in the cytoplasm in a diffuse or granular way (nuclear labeling was not considered).

Inflammatory cells (lymphocytes, their subtypes, and neutrophils) were counted separately in the peritumoral area (in the capsule or connective tissue around the neoplasm) and intratumoral area (in the stroma between the neoplastic lobes and in direct contact with the tumor cells) in the eight photographed hot spots of each sample. Finally, the number of each type of inflammatory cell, in the respective areas, was summed separately for each neoplasm, and the cutoff point to characterize how high and low density of inflammatory infiltrate was based on the median value of each cell in the study population. From the total number of cells, neutrophil/lymphocyte (N/L) and CD79^+^/CD3^+^ lymphocyte ratios were calculated, and the median also served as a cutoff point to separate the samples with high and low expression of inflammatory cells.

All procedures performed in the present study followed the ethical principles for the use of animals in experimentation. The project was approved by the ethics committee on the use of animals (CEUA-UFMG, no. 11/2017).

### 2.4. Statistics

GraphPad Prism software version 8.2 was used for the statistical analysis. Continuous variables (mitotic count and Ki-67 expression) had their normality verified by the Kolmogorov–Smirnov’s test, and the association with the density of inflammatory infiltrate was made by the Mann–Whitney test. For comparison of inflammatory cell infiltrate between different areas on the same tumor, the Wilcoxon test was chosen. Fisher’s exact test was used to analyze the association between qualitative variables (histological types, immunophenotypes, tumor size, presence of lymph node metastasis, and Cox-2 expression) and intensity of inflammation. In all cases, *p* < 0.05 was considered significant. The analysis of overall and specific survival was made by Kaplan–Meier curves and the long-rank test provided the comparison between curves. For the analysis of survival in relation to inflammatory density, the density of each cell type (high and low) was compared between peritumoral and intratumoral areas. Survival was also associated with N/L and CD3^+^/CD79^+^ lymphocyte ratios. The Cox regression model was performed in Jamovi version 2.3.28 for univariate and multivariate analysis, considering variables whose significance was previously established in the survival test by Kaplan–Meier.

## 3. Results

Sixty-one neoplasms with a solid arrangement were analyzed: six (9.8%) malignant myoepitheliomas, eight (13.1%) solid papillary carcinomas, nine (14.8%) carcinomas with a solid pattern, nineteen (31.1%) basaloid carcinomas, and nineteen (31.1%) malignant adenomyoepitheliomas. As for immunophenotypes, 13 neoplasms (21.3%) were luminal A, 42 (68.9%) were luminal B HER-2 negative, and 6 (9.8%) were luminal B HER-2 positive. None of the neoplasms were TN or HER-2 overexpressed.

The ages of the animals in the study ranged from 3 to 19 years old, with a mean age of 11 years old (standard deviation 3.3). The majority were mixed-breed animals (18/61, 29.5%), followed by poodles (16/61, 26.23%), Yorkshire terriers (4/61, 6.56%), and German shepherds and Shih tzus with the same amount (3/61, 4.92%). Other breeds in smaller numbers included dachshunds, French bulldogs, Akitas, Maltese, Rottweilers, and Beagles.

In the quantitative analysis on HE slides, the total number of lymphocytes was significantly higher than that of neutrophils (*p* = 0.003). Most neoplasms, 44/61 (72.1%), presented a greater number of lymphocytes in the peritumoral area than in the intratumoral area (*p* = 0.0001). This was also observed for neutrophils, with infiltration in the peritumoral area being greater than in the intratumoral area in 45/61 samples (73.8%) (*p* = 0.02).

In the count of lymphocyte subtypes after immunostaining for CD3^+^ and CD79^+^, 57 of 61 (93.4%) neoplastic samples showed greater infiltration of total TCD3^+^ cells when compared to BCD79^+^ cells, which was higher in only 4/61(6.6%) samples (*p* = 0.0001). Regarding the location of the infiltrate of TCD3^+^ cells, 37/61 (60.7%) neoplasms presented a higher amount of those cells in the intratumoral area compared to the peritumoral area, but no significant difference was seen (*p* = 0.20). Meanwhile, BCD79^+^ cells were in significantly higher number in the peritumoral area in 47/61 (77.3%) neoplasms than in the intratumoral area (*p* = 0.02).

Median and standard deviation values of each cell type are shown in Table 1. Table 2 and Table 3 contain the morphological and immunophenotypic characteristics of the infiltrate in each subtype of neoplasms.

### 3.1. Association Between Inflammatory Infiltrate in Relation to Subtypes and Immunophenotypes of Solid Neoplasms

**Figure 1:**
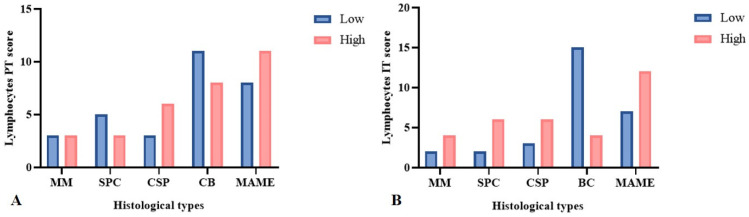
A comparison of the density of TILs in relation to the types of neoplasms with a solid arrangement of the canine mammary gland by Fisher’s exact test. (**A**) The density of the infiltration of peritumoral TILs between histological types. (**B**) The predominance of neoplasms with a high density of intratumoral TILs in MME, SPC, CSP, and MAME, except BC (*p* = 0.02). MME: Malignant myoepithelioma; SPC: solid papillary carcinoma; CSP: carcinoma with a solid pattern; BC: basaloid carcinoma; MAME: malignant adenomyoepithelioma.

**Figure 2:**
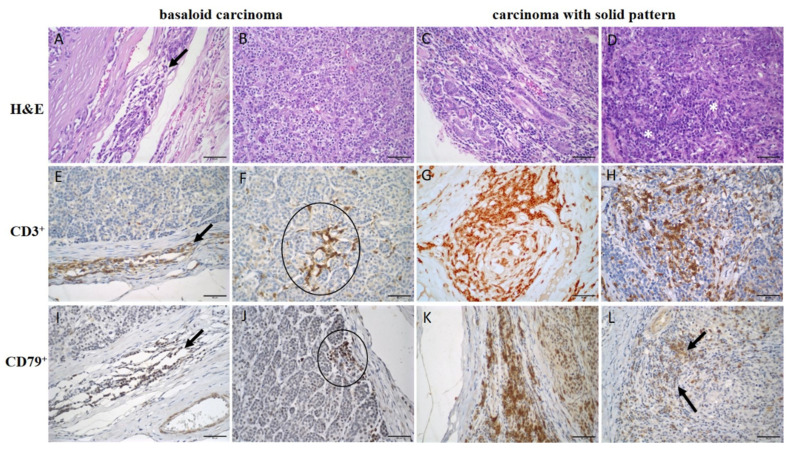
Density of inflammatory infiltrate in basaloid carcinoma compared to carcinoma with solid pattern. (**A**–**D**) TILs on slides stained with H&E (600×); (**A**) Low amount of peritumoral TIL in BC (arrow); (**B**) intratumoral TIL in BC; (**C**) high peritumoral TIL in CSP; (**D**) high intratumoral TIL and forming aggregates in CSP (white asterisk); (**E**–**H**) infiltrate of TCD3^+^ cells in immunohistochemistry (400×); (**E**) low amount of peritumoral lymphocytes in BC (arrow); (**F**) small aggregates of intratumoral lymphocytes in contact with neoplastic cells in BC (circle); (**G**) high infiltration of peritumoral lymphocytes in CSP; (**H**) high infiltration of intratumoral lymphocytes in CSP; (**I**–**L**) infiltrate of TCD79^+^ cells in immunohistochemistry (400×); (**I**) low infiltration of peritumoral lymphocytes in BC (arrow); (**J**) low infiltration of intratumoral lymphocytes in BC (circle); (**K**) high infiltration of peritumoral lymphocytes in CSP; (**L**) high infiltration of intratumoral lymphocytes in CSP (arrow).

In the evaluation of TILs, most CSP (6/66.7%), SPC (6/75%), MME (4/66.7%), and MAME (12/63.2%) neoplasms showed a significantly higher density of TILs in the intratumoral area when compared to BC tumors, among which only 4/21.1% neoplasms presented a high density of TILs (*p* = 0.02). In the peritumoral area, no difference in the density of the lymphocyte infiltrate was observed between histological types (Figure 1A,B). The density of TANs, TCD3^+^, and BCD79^+^ cells in the peri- and intratumoral areas showed no significant differences between histological types (Table 1 and Table 2). Figure 2 shows the intensity of cellular infiltration between basaloid carcinoma and carcinoma with a solid pattern.

**Figure 3:**
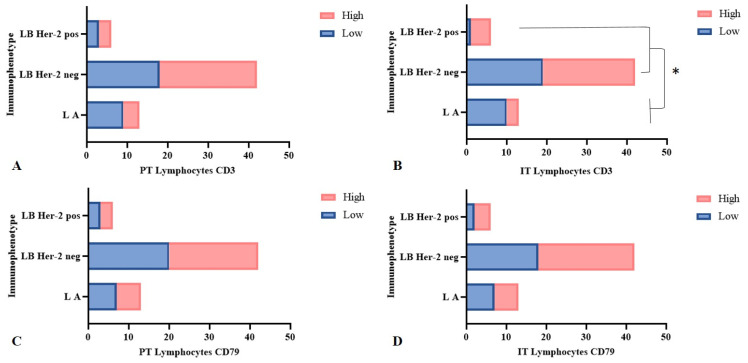
Density of infiltration of TCD3^+^ and BCD79^+^ relative to luminal A, luminal B HER-2 negative, and luminal B HER-2 positive immunophenotypes in solid arrangement neoplasms, Fisher’s exact test. (**A**) Density of peritumoral (PT) TCD3^+^cells (*p* = 0.28); (**B**) luminal B neoplasms having higher density of intratumoral (IT) TCD3^+^ cells compared to luminal A (*p* = 0.04 *); (**C**) density of peritumoral (PT) BCD79^+^ cell infiltration (*p* = 0.92); (**D**) density of intratumoral (IT) BCD79^+^ cells (*p* = 0.71). LA: luminal A; LB: luminal B.

Neoplasms with luminal B immunophenotypes (HER-2 negative or positive) showed higher density of intratumoral TCD3^+^ lymphocytes when compared to luminal A neoplasms (*p* = 0.04). Only three out of thirteen (23.1%) luminal A neoplasms presented a high infiltration of intratumoral TCD3^+^ lymphocytes, while luminal B HER-2 negative or HER-2 positive presented a higher percentage of neoplasms with a high density of this cell type, which were 23/42 (54.8%) and 5/6 (83.3%), respectively. The density of the infiltration of CD79^+^ cells (Figure 3), as TILs and TANs, and their respective locations showed no significant association with immunophenotypes.

### 3.2. Association Between Proliferative Rate, Presence of Lymph Node Metastases, and Inflammatory Infiltrate

**Figure 4:**
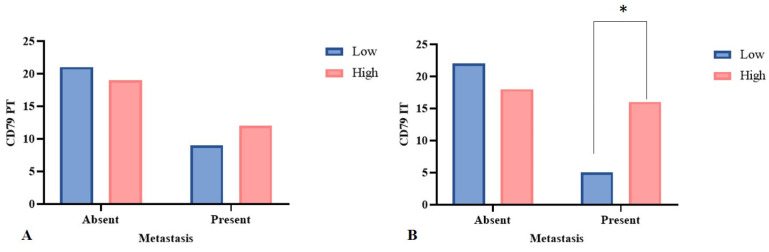
The density of the inflammatory infiltrate in relation to the presence of metastases in regional lymph nodes. Fisher’s exact test. (**A**) The infiltration of peritumoral BCD79^+^ cells showed no difference between neoplasms with or without lymph node metastases (*p* = 0.59). (**B**) The infiltration of intratumoral BCD79^+^ cells was high in female dogs that had metastasis compared to those that did not (*p* = 0.02). * Statistically significant.

Twenty-one female dogs (34.4%) had lymph node metastases. Of these, 16 (76.2%) had a high density of intratumoral BCD79^+^ lymphocyte infiltrate (*p* = 0.03). On the other hand, the density of this peritumoral cell was not associated with the presence of metastasis (*p* = 0.59) (Figure 4). The density of TANs in the peritumoral area was not statistically significantly associated with lymph node metastases (*p* = 0.05), even though 15 (71.9%) of the 21 neoplasms that metastasized to lymph nodes exhibited a TAN density above the median in the peritumoral area. For TILs and TCD3^+^ cells, no significant difference was observed between the density and the site of inflammation and lymph node status.

Neoplasms with a high density of intratumoral TILs had a higher mitotic count with a median of 20 mitotic figures (2.37 mm^2^), while neoplasms with a low density of TILs had a median of 14 mitotic figures (*p* = 0.03). However, the density of peritumoral TILs was not significantly related to the mitotic count (*p* = 0.05). A high density of peritumoral TCD3^+^ and BCD79^+^ cells was also associated with a higher mitotic count. Neoplasms with a high infiltration of peritumoral TCD3^+^ and BCD79^+^ lymphocytes presented a median of 21 mitotic figures, while those with low infiltration presented 13 mitotic figures (*p* = 0.006) for both cell types. Regarding intratumoral infiltration of CD3^+^ and CD79^+^, only intratumoral CD79 lymphocyte infiltration was significantly associated with mitotic count (*p* = 0.02). Neoplasms with a high density of those cells had a median of 19 mitotic figures, and those with low infiltration had 12 mitotic figures (*p* = 0.02). The intensities of peri- (*p* = 0.9) and intratumoral (*p* = 0.7) TANs were not associated with mitotic counting.

**Figure 5:**
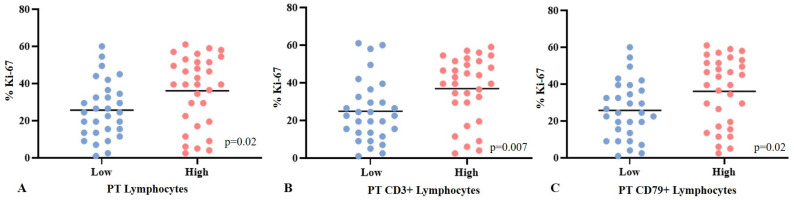
Association of peritumoral inflammation density with immunostaining for Ki-67 in neoplasms with solid arrangement. Mann–Whitney’s test. (**A**–**C**) High density of TILs (*p* = 0.02) and high density of TCD3^+^ cells (*p* = 0.007) and BCD79^+^ cells (*p* = 0.02) are associated with higher percentage of Ki-67. Bar indicates mean.

When evaluating the density of inflammation with Ki-67 immunostaining, we observed that neoplasms with high TILs (*p* = 0.02) and a high infiltration of TCD3^+^ (*p* = 0.007) and BCD79^+^ cells (*p* = 0.02) in the peritumoral area were associated with a higher percentage of immunostaining (Figure 5). Neoplasms with a high density of TILs and TCD3^+^ and BCD79^+^ cells had the same median of Ki-67 labeling (34%), and neoplasms with a low intensity of those cells had a median of 26%, 25%, and 26% of immunostaining, respectively. The remaining locations of TILs and CD3^+^ and CD79^+^ cells, as well as TANs, were not associated with the percentage of Ki-67 labeling.

### 3.3. Association Between Tumor Size and Cox-2 Immunostaining with Inflammatory Infiltrate

The results are detailed in Table 1 and Table 2.

Among the neoplasms evaluated (*n* = 61), 25 (41%) were classified as T1, 12 (19.7%) as T2, and 24 (39.3%) as T3. No association of tumor size with the density of TILs, TANs, TCD3^+^, and BCD79^+^ was observed.

Of the 59 neoplasms evaluated for Cox-2, only 17 (28.8%) presented positive immunostaining; 16 presented scores up to three, and 1 presented a score of six. No statistical difference was seen between peri- and intratumoral inflammations and Cox-2 positivity.

### 3.4. Association Between Inflammatory Infiltrate and Survival Time

Information on overall survival was available for 47/61 (77%) female dogs, of which 35 (74%) died and 12 (26%) were alive at the end of the study. Of the 35 dogs that died, 22 (63%) died from causes related to the mammary tumor. The median overall survival was 365 days (95% CI, 283 to 630 days), and the specific survival was 670 days (95% CI, with a minimum of 365).

Female dogs who had neoplasms with a high density of intratumoral TANs had shorter median overall survival time than those with a low infiltration of such cells (*p* = 0.04, HR = 2.07, CI: 1.02–4.21), which were 210 and 425 days of survival, respectively. On the other hand, infiltration with peritumoral TANs (*p* = 0.2) had no influence on survival. Also, overall survival was not associated with TILs in the peri- (*p* = 0.14) and intratumoral (*p* = 0.10) areas, the infiltration of peri- (*p* = 0.48) and intratumoral (*p* = 0.05) TCD3^+^ cells, and the infiltration of peri- (*p* = 0.17) and intratumoral (*p* = 0.11) BCD79^+^ cells (Table 4).

**Figure 6:**
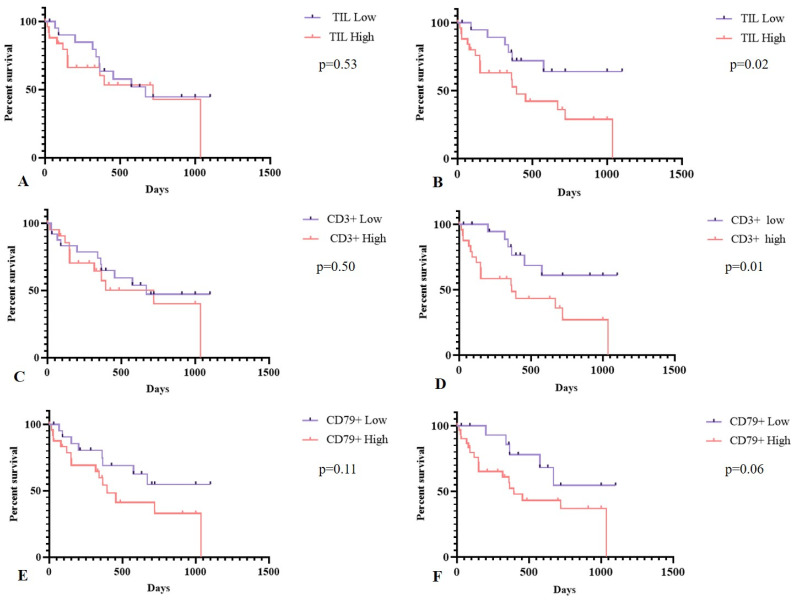
The specific survival of female dogs (*n* = 34; 22 died and 12 alive) with mammary gland neoplasms with a solid arrangement in relation to the presence of TILs, TCD3^+^, and BCD79^+^ cells. Kaplan–Meier. (**A**) The infiltrate of peritumoral (PT) TILs; (**B**) the infiltrate of intratumoral (IT) TILs. (**C**) The infiltrate of peritumoral (PT) TCD3^+^ cells and (**D**) intratumoral (IT) TCD3^+^ cells; (**E**) the infiltrate of peritumoral (PT) B CD79^+^ cells; (**F**) intratumoral (IT) CD79^+^ cells.

In the specific survival evaluation, 50% of female dogs presenting neoplasms with a high density of intratumoral TILs had died in 395 days, and 64% of those with a low density of TILs were still alive after 1000 days (*p* = 0.02, HR = 2.57, CI:1.11–5.97). That was also similar to what was observed in the evaluation of TCD3^+^ cells, where patients with high intratumoral density showed a median survival time of 365 days, and 61% of those with low intratumoral density were still alive after 1000 days (*p* = 0.01, HR = 2.85, CI:1.23–5.63). The density of peritumoral TILs (*p* = 0.53) and TCD3^+^ (*p* = 0.50), peri- (*p* = 0.11) and intratumoral (*p* = 0.06) BCD79^+^ cells, and peri- (*p* = 0.48) and intratumoral TANs (*p* = 0.28) was not associated with dog survival (Figure 6; Table 4).

The N/L and CD3+/CD79 cell ratios were also unrelated to the overall (*p* = 0.54; *p* = 0.53) and specific (*p* = 0.81, *p* = 0.57) survival time of female dogs.

In the specific survival analysis by Cox regression, the univariate analysis showed that a high density of intratumoral TILs increases the risk of death related to the neoplasm. The high presence of TILs is associated with a risk ratio of 2.76 times (*p* = 0.03, confidence interval, CI, 95% = 1.08–7.07), and the high infiltration of intratumoral TCD3^+^ cells increased the risk of death related to the neoplasm by three times (*p* = 0.02, CI:1.19–7.8). Female dogs with metastases to regional lymph nodes also had a risk of death 6.5 (CI: 2.61–16.4) times higher. In the multivariate analysis, the only variable that remained associated with the risk of death was the presence of metastasis in lymph nodes, which was responsible for a three-fold increase in the risk of death from the disease (*p* < 0.001) (Table 5).

## 4. Discussion

In canine neoplasms with a solid arrangement, the characteristics of tumor-infiltrating inflammatory cells were associated with survival time, univariate analysis, and some prognostic factors. The increase in the inflammatory infiltrate of lymphocytes and their subtypes was associated with some poor prognostic factors, such as higher mitotic count, Ki-67 expression, and a higher frequency of metastasis to lymph nodes.

Female dogs presenting neoplasms with a solid arrangement with a high density of intratumoral TILs and TCD3^+^ cells had significantly lower specific survival time when compared to female dogs presenting neoplasms with a low density of intratumoral TILs and CD3^+^ cells. This is in agreement with what is reported in women who also have luminal neoplasms, leading to an association of a high number of TILs with shorter survival time [13]. This is believed to be related to the type of lymphocytes present since mammary neoplasms with luminal immunophenotype have a lower percentage of TCD8^+^ cells when compared to TN neoplasms [11]. TCD8^+^ cells are involved with the anti-tumor response mechanism, leading to neoplastic cell lysis. In contrast, other types of T cells that favor cancer progression and shorten survival may be present, such as regulatory T cells (such as Foxp3) and CD4 helper 2 cells [38,39]. A limitation of this study was not identifying T lymphocyte subtypes, such as CD4^+^ and CD8^+^, or Foxp3. However, we suggest the presence of a predominant immunosuppression and tolerance response in the tumor microenvironment.

The neoplasms with a solid arrangement evaluated were MME (9.8%), SPC (13.1%), CSP (14.8%), BC (31.1%), and MAME (31.1%). We had few neoplasms diagnosed as MME and SPC, which may be a limitation regarding the representativeness of the various histological types of these neoplasms. However, some of them are infrequent, as seen in a study reclassifying 72 cases of solid carcinomas in female dogs, where MME represented only 15% of these neoplasms [40].

There was a significant statistical predominance of lymphocytes in relation to neutrophils in all samples, corroborating other studies that evaluated the inflammatory infiltrate in several histological types of mammary neoplasms in female dogs [18,25]. Lymphocytes are the most studied cells in the tumor microenvironment of several canine neoplasms, such as oral and cutaneous melanomas [41,42], urothelial bladder cell carcinoma [43], and mammary cancer [15,16,18]. However, few authors specify the areas where this inflammation is located [17,33,44].

In canine mammary neoplasms with a solid arrangement, the stroma is often scarce among neoplastic nests, making it difficult to assess stromal lymphocytic inflammation as it is studied in women. Therefore, we evaluated peritumoral and intratumoral inflammation, as was already performed in other studies in veterinary medicine [18,33]. In women’s breast cancer, the evaluation of TILs focusses on the stromal area between neoplastic nests, and the percentage of the area occupied by the inflammation is used in prognostic and predictive analysis [45].

In histology, we identified that most of the evaluated neoplasms show a greater amount of infiltration of TILs in the peritumoral area (72.1%) in relation to the intratumoral area (*p* = 0.0001). In the evaluation of lymphocyte subtypes, the majority of neoplasms (93.4%) showed a significantly higher amount of T lymphocytes than B lymphocytes (*p* = 0.0001), similar to what has already been described in women [46], cats [47], and female dogs [17]. There was no statistical difference in the amount of TCD3 lymphocytes between the peri- and intratumoral areas in the samples, but CD79 B lymphocytes predominated in the peritumoral area (*p* = 0.02). Similar to what is seen in breast cancer in women, TCD3^+^ cells show reasonable homogeneity in samples and may be around and inside the nests of tumor cells, while the CD20^+^ cell, a marker used for B cells in women, is seen in less quantity and often forming peritumoral aggregates [48]. The formation of peritumoral lymphoid aggregates, which consist predominantly of B cells, is reported in in situ and invasive ductal carcinoma of women, indicating a host’s humoral immune response secondary to the neoplastic cell proliferation [49].

BCs are characterized by compact cell nest formations with cells presenting more hyperchromatic nuclei, especially in the periphery, where cells are organized into palisades [30]. This may cause greater difficulty in the visualization of lymphocytes within the tumor nests and may have influenced the lower intensity of intratumoral TILs we found in this histological type compared to the others (*p* = 0.02). Thus, we may infer that the histological and growth patterns of the neoplasm can interfere with the quantification of TILs, as described in women’s neoplasms [45].

As for immunophenotypes, only luminal A, luminal B HER-2 negative, and luminal B HER-2 positive immunophenotypes were diagnosed. In this study, neoplasms with luminal B HER-2 positive and negative immunophenotypes presented significantly higher density of intratumoral TCD3^+^ lymphocyte infiltration when compared to those of luminal A immunophenotype (*p* = 0.04). The mammary cancer immunophenotype is known to influence the amount and composition of inflammatory infiltrate in the tumor microenvironment [50,51]. Canine luminal A neoplasms may present less activation of genes that produce proteins associated with immune mechanism and inflammatory stimulus than B immunophenotype neoplasms [52]. In women, luminal A mammary neoplasms have also been described as presenting a lower presence of TILs than luminal B HER-2 negative neoplasms [53]. Similarly, TN- and HER-2-overexpressed neoplasms also incite greater chemotaxis, both of lymphocytes [14,15,39] and neutrophils [23], due to greater genomic instability.

During the inflammatory process, several growth factors and cytokines that activate transcription factors are produced, such as NF-kB and STAT-3, which favor cell proliferation [54]. This is in agreement with what we observed, i.e., the high intensity of intra- and peritumoral lymphocyte inflammation was observed in neoplasms with a higher mitotic count and/or higher immunostaining of Ki-67. In the present study, a high mitotic count was associated with a high density of intratumoral TILs (*p* = 0.03) and CD79^+^cells (*p* = 0.02). In addition, both the mitotic count and Ki-67 immunostaining were proportionally related to the density of peritumoral CD3^+^ and C79^+^ cells. As cells proliferate, they can expose new antigens and induce a greater inflammatory response, primarily by cells of the innate immune system, such as TCD8 ^+^ and NK lymphocytes.

Metastasis to regional lymph nodes was significantly associated with a high density of intratumoral lymphocytes. This finding corroborates other studies of canine mammary tumors, describing a higher percentage of BCD79^+^ cells infiltrating carcinoma in mixed tumors of animals presenting metastasis to lymph nodes [18] and greater peritumoral lymphatic invasion in several types of mammary carcinomas when compared to tumors with a low infiltration of such cells [16]. In canine melanoma, lymph node metastasis is also related to a moderate amount of intratumoral B cells in the primary neoplasm [42]. In both women and in experimental mouse models, an increased infiltration of B cells was detected in the microenvironment of neoplasms that metastasized to lymph nodes [49,55], and many of those lymphocytes were regulatory B cells, responsible for producing cytokines that reduce the anti-tumor function of T cells via the secretion of IL-10 [49,56]. In experimental models, such an increase in tumor B cells is associated with the appearance of stromal cells in the lymph node and an increase of transcription genes related to important signaling pathways in pre-metastatic nests in lymph nodes [55]. Even though CD79^+^ B lymphocytes were associated with lymph node metastasis in the female dogs of the present study, the high infiltration of those did not influence the overall and specific survival time. However, we observed that the presence of lymph node metastasis increased the risk of death due to the neoplasm by six and three times in univariate and multivariate analysis, respectively, which makes it an independent prognostic factor. Metastasis to lymph nodes was also a factor that increased the risk of cancer-related death in female dogs in other studies [18,35].

Although we did not observe a significant association of neutrophils with the presence of metastasis to lymph nodes, 71,4% of the neoplasms that metastasized had a high density of peritumoral TANs (*p* = 0.05). In addition, intratumoral neutrophil infiltration generated an important difference in the overall survival time of female dogs; those with low intratumoral density had a median survival time of 425 days, while those with a high density of neutrophil infiltrate had a median survival time of 210 days (*p* = 0.04). In human medicine, few breast cancer articles make this association. One of them describes shorter survival time in patients with a high density of neutrophil infiltrate but without specifying the location [24]. To date, no other study associating tumor neutrophil infiltration with the survival of female dogs with mammary neoplasms is known.

Some studies associate a larger tumor size with a greater presence of TILs in women’s luminal neoplasms [50,57], but we did not observe a significant association of this variable with TILs, T cells (CD3^+^), B cells (CD79^+^), and neutrophils.

A higher Cox-2 score was associated with higher tumor inflammatory infiltrate in mammary neoplasms [58] and canine melanoma [41]. However, that finding was not observed in the present study. This may have occurred because we had only 28.8% of neoplasms marked for Cox-2, and most of them presented low scores. Cyclooxygenase is probably not an important factor for inflammatory stimulation in neoplasms with a solid arrangement; however, further studies should be conducted to confirm this hypothesis.

The blood neutrophil/lymphocyte ratio has been performed in patients with mammary carcinomas and is indicated as a prognostic factor in studies of cats [59]. In female dogs, a blood ratio >5 N/L before surgery is associated with worse prognosis [60]. Here, we used the cell count in the tumor microenvironment to associate the ratio of N/L and CD79^+^/CD3^+^ with survival, but it was not significant.

In a study of mammary neoplasms in female dogs, the animals presenting neoplasms with greater lymphocyte infiltration (>600 lymphocytes/8 fields) had lower survival time, which was confirmed by the multivariate Cox regression analysis [18]. In the univariate analysis, we observed that the high density of intratumoral TILs and intratumoral T lymphocytes increased the risk of death from the neoplasm by 2.7 and 3 times, respectively, but this was not confirmed by the multivariate analysis.

We believe that by identifying the type of inflammatory cells present in these canine mammary tumors and understanding their influence on neoplastic progression, new therapeutic measures targeting immune system modulation can be researched and implemented. Furthermore, it will be possible to correlate the density and location of TILs with the response to chemotherapy, as described in women, where a higher quantity of TILs has been associated with a better response to chemotherapy [13,14,39].

## 5. Conclusions

In our study, we observed that neoplasms with a solid arrangement show a greater predominance of lymphocytes than neutrophils and mainly TCD3^+^ cells. TCD3^+^ cells predominated in the intratumoral area, while BCD79^+^ cells predominated in the peritumoral area.

The infiltration of lymphocytes and their location in canine mammary neoplasms, evaluated here, are associated with some prognostic factors in female dogs. Nodal metastasis was associated with a high infiltration of intratumoral CD79^+^ cells, and shorter survival time was associated with a high density of TILs and intratumoral T CD3^+^ cells. Thus, lymphocyte infiltration, especially the intratumoral infiltration, may be an important feature in cancer progression, inciting a pro-tumor and regulatory response of the anti-tumor response and influencing the survival of female dogs with mammary neoplasms with a solid arrangement. However, in this study, it was not an independent prognostic factor.

We know that in the laboratory routine, the quantitative evaluation of inflammatory cells in the tumor microenvironment is difficult to carry out. However, the analysis of the intensity of peri- and intratumoral inflammation from a cutoff point can be performed in hematoxylin and eosin stains and associated with morphological characteristics to gather possible prognostic information.

## Figures and Tables

**Table 1:** Counts of lymphocyte and neutrophil (H&E) and CD3^+^ and CD79^+^ cells (immunohistochemistry) in canine mammary neoplasms with solid arrangement (*n* = 61).

	L PT	L IT	N PT	N IT	CD3^+^ PT	CD3^+^ IT	CD79^+^ PT	CD79^+^ IT
Mean	305	185	13.9	11.0	258	302	122	84.2
Median	221	100	8	5	202	215	73	24
Standard deviation	295	301	18.1	22.9	214	287	160	130

n: sample number; L: Lymphocytes; PT: peritumoral; IT: intratumoral.

**Table 2:** Morphological and immunophenotypic characteristics and frequency of infiltration density of lymphocytes and neutrophils in canine mammary neoplasms with solid arrangement (*n* = 61). Fisher’s exact test.

Variables	Lymphocytes						Neutrophils					
	PT		*p*	IT		*p*	PT		*p*	IT		*p*
	Low	High		Low	High		Low	High		Low	High	
***Histhological types***												
CSP	3 (33.3%)	6 (66.7%)	0.68	3 (33.3%)	6 (66.7%)	0.02 *	2 (22.2%)	7 (77.8%)	0.43	4 (44.4%)	5 (55.6%)	0.68
SPC	5 (62.5%)	3 (37.5%)		2 (25%)	6 (75%)		4 (50%)	4 (50%)		3 (37.5%)	5 (62.5%)	
BC	11 (57.9%)	8 (42.1%)		15 (78.9%)	4 (21.1%)		9 (47.4%)	10 (52.6%)		12 (63.2%)	7 (36.8%)	
MM	3 (50%)	3 (50%)		2 (33.3%)	4 (66.7%)		4 (66.7%)	2 (33.3%)		3 (50%)	3 (50%)	
MAME	8 (42.1%)	31 (57.9%)		7 (37.8%)	12 (63.2%)		11 (57.9%)	8 (42.1%)		8 (42.1%)	11 (57.9%)	
***Immunophenotype***												
L A	8 (61.5%)	5 (38.5%)	0.47	4 (30.8%)	9 (69.2%)	0.26	9 (69.2%)	4 (30.8%)	0.1	8 (61.5%)	5 (38.5%)	0.2
L B HER-2 -	20 (47.6%)	22 (52.4%)		23 (54.8%)	19 (45.2%)		20 (47.6%)	22 (52.4%)		21 (50%)	21 (50%)	
L B HER-2 +	2 (33.3%)	4 (66.7%)		2 (33.3%)	4 (66.7%)		1 (16.7%)	5 (83.3%)		2 (33.3%)	4 (66.7%)	
***Size (T)***												
1 (1–3 cm)	14 (56%)	11 (44%)	0.67	16 (64%)	9 (36%)	0.09	12 (48%)	13 (52%)	1.0	14 (56%)	11 (44%)	0.67
2 (3–5 cm)	5 (41.7%)	7 (58.3%)		4 (33.3%)	8 (66.7%)		6 (50%)	6 (50%)		5 (41.7%)	7 (58.3%)	
3 (>5 cm)	11 (45.8%)	13 (54.2%)		9 (37.5%)	15 (62.5%)		12 (50%)	12 (50%)		11 (45.8%)	13 (54.2%)	
***Nodal Metastasis***												
Present	7 (33.3%)	14 (66.7%)	0.1	11 (52.4%)	10 (47.6%)	0.79	6 (28.6%)	15 (71.4%)	0.05	8 (38.1%)	13 (61.9%)	0.28
Absent	23 (57.5%)	17 (42.5%)		19 (47.5%)	21 (52.5%)		23 (57.5%)	17 (42.5%)		22 (55%)	18 (45%)	
***Cox-2***												
negative	26 (63.4%)	15 (36.6%)	0.55	23 (54.8%)	19 (45.2%)	0.25	21 (50%)	21 (50%)	0.57	23 (54.8%)	19 (45.2%)	0.09
positive	9 (52.9%)	8 (47.1%)		6 (35.3%)	11 (64.7%)		7 (41.2%)	10 (58.8%)		5 (29.4%)	12 (70.6%)	

PT: peritumoral; IT: intratumoral; CSP: carcinoma with solid pattern; SPC:solid papillary carcinoma; BC: basaloid carcinoma; MAME: malignant adenomyoepithelioma; MME: malignant myoepithelioma L: luminal; *statistically significant when *p* < 0.05.

**Table 3:** Morphological and immunophenotypic characteristics and frequency of infiltration density of TCD3^+^ and BCD79^+^ cells in canine mammary neoplasms with solid arrangement (*n* = 61). Fisher’s exact test.

Variables	Lymphocytes CD3						Lymphocytes CD79					
	PT		*p*	IT		*p*	PT		*p*	IT		*p*
	Low	High		Low	High		Low	High		Low	High	
***Histhological types***												
CSP	3 (33.3%)	6 (66.7%)	0.65	3 (33.3%)	6 (66.7%)	0.23	3 (33.3%)	6 (66.7%)	0.68	1 (11.1%)	8 (88.9%)	0.19
SPC	5 (62.5%)	3 (37.5%)		2 (25%)	6 (75%)		5 (62.5%)	3 (37.5%)		4 (50%)	4 (50%)	
BC	8 (42.1%)	11 (57.9%)		10 (52.6%)	9 (47.4%)		8 (42.1%)	11 (57.9%)		11 (57.9%)	8 (42.1%)	
MM	4 (66.7%)	2 (33.3%)		5 (83.3%)	1 (16.7%)		3 (50%)	3 (50%)		2 (33.3%)	4 (66.7%)	
MAME	10 (52.6%)	9 (47.4%)		10 (52.6%)	9 (47.4%)		11 (57.9%)	8 (42.1%)		9 (47.9%)	10 (52.6%)	
***Immunophenotype***												
L A	9 (69.2%)	4 (30.8%)	0.28	10 (76.9%)	3 (23.1%)	0.04 *	7 (53.8%)	6 (46.2%)	0.92	7 (53.8%)	6 (46.2%)	0.71
L B HER-2 -	18 (42.9%)	24 (57.1%)		19 (45.2%)	23 (54.8%)		20 (47.6%)	22 (52.4%)		18 (42.9%)	24 (57.1%)	
L B HER-2 +	3 (30%)	3 (50%)		1 (16.7%)	5 (83.3%)		3 (50%)	3 (50%)		2 (33.3%)	4 (66.7%)	
***Size (T)***												
1 (1–3 cm)	12 (48%)	13 (52%)	0.84	11 (44%)	14 (56%)	0.56	14 (56%)	11 (44%)	0.67	13 (52%)	12 (48%)	0.31
2 (3–5 cm)	5 (41.7%)	7 (58.3%)		5 (41.7%)	7 (58.3%)		5 (41.7%)	7 (58.3%)		3 (25%)	9 (75%)	
3 (>5 cm)	13 (54.2%)	11 (45.8%)		14 (58.3%)	10 (41.7%)		11 (45.8%)	13 (54.2%)		11 (45.8%)	13 (54.2%)	
***Nodal Metastasis***												
Present	11 (52.4%)	10 (47.6%)	0.79	7 (33.3%)	14 (66.7%)	0.1	21 (52.5%)	19 (47.5%)	0.59	5 (23.8%)	16 (76.2%)	0.03 *
Absent	19 (47.5%)	21 (52.5%)		23 (57.5%)	17 (42.5%)		9 (42.9%)	12 (57.1%)		22 (55%)	18 (45%)	
***Cox-2***												
negative	19 (45.2%)	23 (54.8%)	0.25	21 (50%)	21 (50%)	1.0	19 (45.2%)	23 (54.8%)	0.25	22 (52.4%)	20 (47.6%)	0.15
positive	11 (64.7%)	6 (35.3%)		9 (52.9%)	8 (47.1%)		11 (64.7%)	6 (35.3%)		5 (29.4%)	12 (70.6%)	

PT: peritumoral; IT: intratumoral; CSP: carcinoma with solid pattern; SPC:solid papillary carcinoma; BC: basaloid carcinoma; MAME: malignant adenomyoepithelioma; MME: malignant myoepithelioma L: luminal; *statistically significant when *p* < 0.05.

**Table 4:** The median survival time of female dogs with neoplasms with a solid arrangement and its association with the density of lymphocyte infiltrate, neutrophils, TCD3^+^, and TCD79^+^ cells.

Cell Infiltration	Median Overall Survival (Days)					
	Peritumoral		*p*	Intratumoral		*p*
	Low	High		Low	High	
Lymphocytes	455	324	0.14	425	321	0.1
Neutrophils	395	365	0.2	425	210	0.04 *
CD3 Lymphocytes	395	341	0.48	425	360	0.05
CD79 Lymphocytes	500	365	0.17	575	318	0.11
	**Median specific survival (days)**					
Lymphocytes	670	720	0.53	------	395	0.02 *
Neutrophils	670	575	0.48	1035	575	0.28
CD3 Lymphocytes	670	720	0.5	------	365	0.01 *
CD79 Lymphocytes	------	395	0.11	------	395	0.06

* statistically significant when *p* < 0.05. Long-rank test. ------ more than 50% of animals were still alive at the end of the study.

**Table 5:** Univariate and multivariate analyses of specific survival (Cox model) in relation to intratumoral TIL and CD3^+^ in female dogs with neoplasms with solid arrangement.

*Variables*		HR	95% CI	*p*-Value
		Univariate Analyses		
Metastasis	Absent			
	Present	6.54	2.61–16.4	<0.001 *
IT Lymphocytes	Low			
	High	2.76	1.08–7.07	0.03 *
IT CD3 Lymphocytes	Low			
	High	3.05	1.19–7.80	0.02 *
		Multivariate analyses		
Metastasis	Absent			
	Present	3.23	1.09–9.57	0.03 *
IT Lymphocytes	Low			
	High	0.73	0.22–2.39	0.6
IT CD3 Lymphocytes	Low			
	High	0.82	0.25–2.72	0.7

HR, hazard ratio, CI, Confidence interval, IT: intratumoral; TILs: tumor-infiltrating lymphocytes. * Statistically significant when *p* < 0.05.

## Data Availability

Data are contained within the article and Supplementary Materials.

## Supplementary Materials

The following supporting information can be downloaded at https://www.mdpi.com/article/10.3390/ani15020287/s1, Figure S1. Canine Mammary Neoplasms with Solid Arrangement. Malignant Adenomyoepithelioma: A. Neoplastic cells were organized in mantles or solid nests, with scarce tubules and stroma, HE, bar = 100 µm. B. Immunolabelling for pancytokeratin in the cytoplasm of epithelial cells, bar = 50 µm. C. Immunolabelling for p63 in the nuclei of myoepithelial cells, bar = 50 µm. Carcinomas with Solid Pattern: D. Neoplastic cells were organized in solid nests, HE, bar = 100 µm. E. Immunolabelling for pancytokeratin in the cytoplasm of epithelial cells, bar = 50 µm. F. No immunostaining for p63, bar = 50 µm. Basaloid carcinoma: G. solid nests surrounded by a layer of peripheral palisading cells (arrow head), multinodular pattern separated by moderate fibrous stroma (*), HE, bar = 100 µm. H. Central cells immunolabelled for pancytokeratin, bar = 50 µm. I. Palisading peripheral cells stained with cytokeratin 14 (arrow), bar = 50 µm. Malignant Myoepithelioma: J. Elongated and rounded cells oriented in various directions, HE, bar = 100 µm. K. Cells with clear cytoplasm, round nuclei, and intercellular myxoid material (*), HE, bar = 50 µm. L. Immunolabelling for p63 in the nuclei of myoepithelial cells, bar = 50 µm. Solid Papillary Carcinomas: M. Papillary projections with multiple layers of epithelial cells extending from the stroma (arrow), forming a solid appearance, HE, bar = 100 µm. N. Delicate fibrous stroma (arrow) supporting the layers of epithelial cells, Gomori’s trichrome, bar = 50 µm. O. Immunolabelling for pancytokeratin in the cytoplasm of epithelial cells, bar = 50 µm. Figure S2. Immunohistochemistry for estrogen receptor in Canine Mammary Neoplasms with Solid Arrangement. A. Negative immunostaining (<1%). B. Nuclear staining in a few cells (1–25% of labeled cells) (arrow). C. 26–50% of labeled cells. D. 51–75% of labeled cells. E. More than 75% of labeled cells. F. Positive control in the mammary gland of a non-neoplastic female dog. Bar = 50 um. Figure S3. Immunohistochemistry for progesterone receptor in Canine Mammary Neoplasms with Solid Arrangement. A. Negative immunostaining (<1%). B. Nuclear staining in a few cells (1–25% of labeled cells) (arrow). C. 26–50% of labeled cells. D. 51–75% of labeled cells. E. More than 75% of labeled cells. F. Positive control in the mammary gland of a non-neoplastic female dog. Bar = 50 um. Figure S4. Immunohistochemistry for Ki-67 in Canine Mammary Neoplasms with Solid Arrangement. A. Neoplasm with a high nuclear staining count for Ki-67, showing >20% of cells with nuclear staining. B. Neoplasm with a low nuclear staining count for Ki-67, showing <20% of cells with nuclear staining. Bar = 50 um. Figure S5. Immunohistochemistry for HER-2 in Canine Mammary Neoplasms with Solid Arrangement followed the guidelines of membrane marking score (Wolff et al., 2013) [36]. A. Neoplasm with score 1. B. Neoplasm with score 2. C. Neoplasm with score 3. Bar = 50 um. Figure S6. Immunohistochemistry for COX-2 in Canine Mammary Neoplasms with Solid Arrangement, based on a semi-quantitative evaluation of labeled tumor cells in the cytoplasm (Lavalle et al., 2009) [37]. A. Neoplasm without immunostaining for COX-2. B. Neoplasm with a score of 1 (less than 10% of cells labeled; weak intensity) (arrow). C. Neoplasm with a score of 3 (less than 10% of cells labeled; strong intensity) (arrow). Bar = 50 µm.

## References

[1] Klintrup K., Mäkinen J.M., Kauppila S., Väre P.O., Melkko J., Tuominen H., Tuppurainen K., Mäkelä J., Karttunen T.J., Mäkinen M.J. (2005). Inflammation and prognosis in colorectal cancer. Eur. J. Cancer.

[2] Mlecnik B., Tosolini M., Kirilovsky A., Berger A., Bindea G., Meatchi T., Bruneval P., Trajanoski Z., Fridman W.-H., Pagès F. (2011). Histopathologic-Based Prognostic Factors of Colorectal Cancers Are Associated With the State of the Local Immune Reaction. J. Clin. Oncol..

[3] Galdiero M.R., Bianchi P., Grizzi F., Di Caro G., Basso G., Ponzetta A., Bonavita E., Barbagallo M., Tartari S., Polentarutti N. (2016). Occurrence and significance of tumor-associated neutrophils in patients with colorectal cancer. Int. J. Cancer.

[4] Shimizu S., Hiratsuka H., Koike K., Tsuchihashi K., Sonoda T., Ogi K., Miyakawa A., Kobayashi J., Kaneko T., Igarashi T. (2019). Tumor-infiltrating CD8^+^ T-cell density is an independent prognostic marker for oral squamous cell carcinoma. Cancer Med..

[5] Stravodimou A., Tzelepi V., Papadaki H., Mouzaki A., Georgiou S., Melachrinou M., Kourea E.P. (2018). T-lymphocytes and their subpopulations in invasive and in situ squamous cell carcinoma of the skin and actinic keratosis. J. Cutan. Pathol..

[6] Shen M., Hu P., Donskov F., Wang G., Liu Q., Du J. (2014). Tumor-Associated Neutrophils as a New Prognostic Factor in Cancer: A Systematic Review and Meta-Analysis. PLoS ONE.

[7] Zhu S., Lin J., Qiao G., Xu Y., Zou H. (2015). Differential regulation and function of tumor-infiltrating T cells in different stages of breast cancer patients. Tumor Biol..

[8] Yu X., Zhang Z., Wang Z., Wu P., Qiu F., Huang J. (2015). Prognostic and predictive value of tumor-infiltrating lymphocytes in breast cancer: A systematic review and meta-analysis. Clin. Transl. Oncol..

[9] Liu K., Zhao K., Wang L., Sun E. (2018). The prognostic values of tumor-infiltrating neutrophils, lymphocytes and neutrophil/lymphocyte rates in bladder urothelial cancer. Pathol.-Res. Pract..

[10] Denkert C., Loibl S., Noske A., Roller M., Müller B.M., Komor M., Budczies J., Darb-Esfahani S., Kronenwett R., Hanusch C. (2010). Tumor-Associated Lymphocytes As an Independent Predictor of Response to Neoadjuvant Chemotherapy in Breast Cancer. J. Clin. Oncol..

[11] Mahmoud S.M., Paish E.C., Powe D.G., Macmillan R.D., Grainge M.J., Lee A.H., Ellis I.O., Green A.R. (2011). Tumor-Infiltrating CD8^+^ Lymphocytes Predict Clinical Outcome in Breast Cancer. J. Clin. Oncol..

[12] A Mohammed Z.M., Going J.J., Edwards J., Elsberger B., McMillan D.C. (2013). The relationship between lymphocyte subsets and clinico-pathological determinants of survival in patients with primary operable invasive ductal breast cancer. Br. J. Cancer.

[13] Gao Z.-H., Li C.-X., Liu M., Jiang J.-Y. (2020). Predictive and prognostic role of tumour-infiltrating lymphocytes in breast cancer patients with different molecular subtypes: A meta-analysis. BMC Cancer.

[14] Lee H.J., Kim J.Y., Park I.A., Song I.H., Yu J.H., Ahn J.-H., Gong G. (2015). Prognostic Significance of Tumor-Infiltrating Lymphocytes and the Tertiary Lymphoid Structures in HER2-Positive Breast Cancer Treated With Adjuvant Trastuzumab. Am. J. Clin. Pathol..

[15] Wang K., Xu J., Zhang T., Xue D. (2016). Tumor-infiltrating lymphocytes in breast cancer predict the response to chemotherapy and survival outcome: A meta-analysis. Oncotarget.

[16] Kim J.-H., Chon S.-K., Im K.-S., Kim N.-H., Sur J.-H. (2013). Correlation of tumor-infiltrating lymphocytes to histopathological features and molecular phenotypes in canine mammary carcinoma: A morphologic and immunohistochemical morphometric study. Can. J. Vet. Res..

[17] Muscatello L., Avallone G., Brunetti B., Bacci B., Foschini M., Sarli G. (2022). Standardized approach for evaluating tumor infiltrating lymphocytes in canine mammary carcinoma: Spatial distribution and score as relevant features of tumor malignancy. Vet. J..

[18] Estrela-Lima A., Araújo M.S., Costa-Neto J.M., Teixeira-Carvalho A., Barrouin-Melo S.M., Cardoso S.V., A Martins-Filho O., Serakides R., Cassali G.D. (2010). Immunophenotypic features of tumor infiltrating lymphocytes from mammary carcinomas in female dogs associated with prognostic factors and survival rates. BMC Cancer.

[19] Abadie J., Nguyen F., Loussouarn D., Peña L., Gama A., Rieder N., Belousov A., Bemelmans I., Jaillardon L., Ibisch C. (2017). Canine invasive mammary carcinomas as models of human breast cancer. Part 2: Immunophenotypes and prognostic significance. Breast Cancer Res. Treat..

[20] Queen M.M., Ryan R.E., Holzer R.G., Keller-Peck C.R., Jorcyk C.L. (2005). Breast Cancer Cells Stimulate Neutrophils to Produce Oncostatin M: Potential Implications for Tumor Progression. Cancer Res..

[21] Timaxian C., Vogel C.F.A., Orcel C., Vetter D., Durochat C., Chinal C., Nguyen P., Aknin M.-L., Mercier-Nomé F., Davy M. (2021). Pivotal Role for Cxcr2 in Regulating Tumor-Associated Neutrophil in Breast Cancer. Cancers.

[22] Rodriguez G.V., Abrahamsson A., Jensen L.D.E., Dabrosin C. (2017). Estradiol Promotes Breast Cancer Cell Migration via Recruitment and Activation of Neutrophils. Cancer Immunol. Res..

[23] Soto-Perez-De-Celis E., Chavarri-Guerra Y., Leon-Rodriguez E., Gamboa-Dominguez A. (2017). Tumor-Associated Neutrophils in Breast Cancer Subtypes. Asian Pac. J. Cancer Prev..

[24] Kwantwi L.B., Wang S., Zhang W., Peng W., Cai Z., Sheng Y., Xiao H., Wang X., Wu Q. (2021). Tumor-associated neutrophils activated by tumor-derived CCL20 (C-C motif chemokine ligand 20) promote T cell immunosuppression via programmed death-ligand 1 (PD-L1) in breast cancer. Bioengineered.

[25] de Souza T.A., de Campos C.B., De Biasi Bassani Goncalves A., Nunes F.C., Monteiro L.N., de Oliveira Vasconcelos R., Cassali G.D. (2018). Relationship between the inflammatory tumor microenvironment and different histologic types of canine mammary tumors. Res. Vet. Sci..

[26] Flecher M.C., Balabram D., Salles Y.A., Souza F.R., Estrela-Lima A., Nakagaki K.Y.R., Cassali G.D. (2024). Evaluation of im-munophenotype and inflammation in canine mammary neoplasms with solid arrangement. J. Comp. Pathol..

[27] Zappulli V., Pena L., Rasotto R., Goldschmidt M.H., Gama A., Scruggs J.L., Kiupel M. (2019). Volume 2: Mammary Tumors. Surgical Pathology of Tumors of Domestic Animals Kiupel, M., Ed..

[28] Cassali G., Jark P., Gamba C., Damasceno K., Estrela-Lima A., Nardi A., Ferreira E., Horta R., Firmo B., Sueiro F. (2020). Consensus Regarding the Diagnosis, Prognosis and Treatment of Canine and Feline Mammary Tumors—2019. Braz. J. Vet. Pathol..

[29] Rasotto R., Berlato D., Goldschmidt M.H., Zappulli V. (2017). Prognostic Significance of Canine Mammary Tumor Histologic Subtypes: An Observational Cohort Study of 229 Cases. Vet. Pathol..

[30] Nakagaki K.Y., Nunes M.M., Garcia A.P.V., Nunes F.C., Schmitt F., Cassali G.D. (2022). Solid Carcinoma of the Canine Mammary Gland: A Histological Type or Tumour Cell Arrangement?. J. Comp. Pathol..

[31] Elston  C.W., Ellis I.O., Elston C.W., Ellis I.O. (1998). Assessment of histological grade. Systemic Pathology.

[32] Owen L.N. (1980). TNM Classification of Tumours in Domestic Animals.

[33] Vieira T.C., Oliveira E.A., dos Santos B.J., Souza F.R., Veloso E.S., Nunes C.B., Del Puerto H.L., Cassali G.D. (2022). COX-2 expression in mammary invasive micropapillary carcinoma is associated with prognostic factors and acts as a potential therapeutic target in comparative oncology. Front. Veter.-Sci..

[34] Hammond M.E.H., Hayes D.F., Dowsett M., Allred D.C., Hagerty K.L., Badve S., Fitzgibbons P.L., Francis G., Goldstein N.S., Hayes M. (2010). American Society of Clinical Oncology/College of American Pathologists Guideline Recommendations for Immunohistochemical Testing of Estrogen and Progesterone Receptors in Breast Cancer. J. Clin. Oncol..

[35] Nunes F., Bertagnolli A., Lavalle G., Silveira T., Balabram D., Cassali G. (2022). The prognostic significance of immunophenotypes in canine malignant mammary tumors. Arq. Bras. Med. Vet.-E Zootec..

[36] Wolff A.C., Hammond M.E.H., Hicks D.G., Dowsett M., McShane L.M., Allison K.H., Allred D.C., Bartlett J.M.S., Bilous M., Fitzgibbons P. (2013). Recommendations for Human Epidermal Growth Factor Receptor 2 Testing in Breast Cancer: American Society of Clinical Oncology/College of American Pathologists Clinical Practice Guideline Update. J. Clin. Oncol..

[37] Lavalle G.E., Bertagnolli A.C., Tavares W.L.F., Cassali G.D. (2009). Cox-2 Expression in Canine Mammary Carcinomas. Vet. Pathol..

[38] Huang Y., Ma C., Zhang Q., Ye J., Wang F., Zhang Y., Hunborg P., Varvares M.A., Hoft D.F., Hsueh E.C. (2015). CD4+ and CD8+ T cells have opposing roles in breast cancer progression and outcome. Oncotarget.

[39] Koletsa T., Kotoula V., Koliou G.-A., Manousou K., Chrisafi S., Zagouri F., Sotiropoulou M., Pentheroudakis G., Papoudou-Bai A., Christodoulou C. (2020). Prognostic impact of stromal and intratumoral CD3, CD8 and FOXP3 in adjuvantly treated breast cancer: Do they add information over stromal tumor-infiltrating lymphocyte density?. Cancer Immunol. Immunother..

[40] Yoshimura H., Nakahira R., Kishimoto T.E., Michishita M., Ohkusu-Tsukada K., Takahashi K. (2014). Differences in Indicators of Malignancy Between Luminal Epithelial Cell Type and Myoepithelial Cell Type of Simple Solid Carcinoma in the Canine Mammary Gland. Vet. Pathol..

[41] Gregório H., Raposo T.P., Queiroga F.L., Prada J., Pires I. (2016). Investigating associations of cyclooxygenase-2 expression with angiogenesis, proliferation, macrophage and T-lymphocyte infiltration in canine melanocytic tumours. Melanoma Res..

[42] Porcellato I., Silvestri S., Menchetti L., Recupero F., Mechelli L., Sforna M., Iussich S., Bongiovanni L., Lepri E., Brachelente C. (2019). Tumour-infiltrating lymphocytes in canine melanocytic tumours: An investigation on the prognostic role of CD3^+^ and CD20^+^ lymphocytic populations. Vet. Comp. Oncol..

[43] Inoue A., Maeda S., Kinoshita R., Tsuboi M., Yonezawa T., Matsuki N. (2017). Density of tumor-infiltrating granzyme B-positive cells predicts favorable prognosis in dogs with transitional cell carcinoma. Vet. Immunol. Immunopathol..

[44] Carvalho M.I., Pires I., Prada J., Queiroga F.L. (2011). T-lymphocytic infiltrate in canine mammary tumours: Clinic and prognostic implications. In Vivo..

[45] Salgado R., Denkert C., Demaria S., Sirtaine N., Klauschen F., Pruneri G., Wienert S., Van den Eynden G., Baehner F.L., Penault-Llorca F. (2015). The evaluation of tumor-infiltrating lymphocytes (TILs) in breast cancer: Recommendations by an International TILs Working Group 2014. Ann. Oncol..

[46] Gomez-Μacias G.S., Molinar-Flores G., Lopez-Garcia C.A., Santuario-Facio S., Decanini-Arcaute H., Valero-Elizondo J., Treviño-Alvarado V., Ortiz-Lopez R., Dono A., Esteban-Zubero E. (2020). Immunotyping of tumor-infiltrating lymphocytes in triple-negative breast cancer and genetic characterization. Oncol. Lett..

[47] Nascimento C., Gameiro A., Correia J., Ferreira J., Ferreira F. (2022). The Landscape of Tumor-Infiltrating Immune Cells in Feline Mammary Carcinoma: Pathological and Clinical Implications. Cells.

[48] Mani N.L., Schalper K.A., Hatzis C., Saglam O., Tavassoli F., Butler M., Chagpar A.B., Pusztai L., Rimm D.L. (2016). Quantitative assessment of the spatial heterogeneity of tumor-infiltrating lymphocytes in breast cancer. Breast Cancer Res..

[49] Ishigami E., Sakakibara M., Sakakibara J., Masuda T., Fujimoto H., Hayama S., Nagashima T., Sangai T., Nakagawa A., Nakatani Y. (2018). Coexistence of regulatory B cells and regulatory T cells in tumor-infiltrating lymphocyte aggregates is a prognostic factor in patients with breast cancer. Breast Cancer.

[50] Glajcar A., Szpor J., Hodorowicz-Zaniewska D., Tyrak K.E., Okoń K. (2019). The composition of T cell infiltrates varies in primary invasive breast cancer of different molecular subtypes as well as according to tumor size and nodal status. Virchows Arch..

[51] Zhu B., Tse L.A., Wang D., Koka H., Zhang T., Abubakar M., Lee P., Wang F., Wu C., Tsang K.H. (2019). Immune gene expression profiling reveals heterogeneity in luminal breast tumors. Breast Cancer Res..

[52] Bergholtz H., Lien T., Lingaas F., Sørlie T. (2022). Comparative analysis of the molecular subtype landscape in canine and human mammary gland tumors. J. Mammary Gland. Biol. Neoplasia.

[53] Criscitiello C., Vingiani A., Maisonneuve P., Viale G., Curigliano G. (2020). Tumor-infiltrating lymphocytes (TILs) in ER+/HER2− breast cancer. Breast Cancer Res. Treat..

[54] Shrihari T. (2017). Dual role of inflammatory mediators in cancer. Ecancer.

[55] Gu Y., Liu Y., Fu L., Zhai L., Zhu J., Han Y., Jiang Y., Zhang Y., Zhang P., Jiang Z. (2019). Tumor-educated B cells selectively promote breast cancer lymph node metastasis by HSPA4-targeting IgG. Nat. Med..

[56] Tao H., Lu L., Xia Y., Dai F., Wang Y., Bao Y., Lundy S.K., Ito F., Pan Q., Zhang X. (2014). Antitumor effector B cells directly kill tumor cells via the Fas/FasL pathway and are regulated by IL-10. Eur. J. Immunol..

[57] Murazawa C., Hashimoto N., Kuraishi K., Motoyama M., Hashimoto S.-I., Ikeuchi M., Norimura S., Matsunaga T., Teramoto K., Haba R. (2023). Status and prognostic value of immunological biomarkers of breast cancer. Oncol. Lett..

[58] Carvalho M.I., Pires I., Prada J., Raposo T.P., Gregório H., Lobo L., Queiroga F.L. (2016). High COX-2 expression is associated with increased angiogenesis, proliferation and tumoural inflammatory infiltrate in canine malignant mammary tumours: A multivariate survival study. Vet. Comp. Oncol..

[59] Petrucci G.N., Lobo L., Queiroga F., Martins J., Prada J., Pires I., Henriques J. (2021). Neutrophil-to-lymphocyte ratio is an independent prognostic marker for feline mammary carcinomas. Vet. Comp. Oncol..

[60] Uribe-Querol E., Romero-Romero L., Govezensky T., Rosales C. (2023). Neutrophil to lymphocyte ratio and principal component analysis offer prognostic advantage for dogs with mammary tumors. Front. Vet. Sci..

