# Supplementary material for: Tumor-Infiltrating Lymphocytes and Neutrophils and Their Location in Canine Mammary Neoplasms with a Solid Arrangement: A Prognostic Factor?
Source: Animals (Basel). 2025 Jan 20;15(2):287. doi: 10.3390/ani15020287 (PMC11762997; doi:10.3390/ani15020287)
Supplement: Supplementary file 1 [file animals-15-00287-s001.zip › animals-3340002-supplementary.pdf]

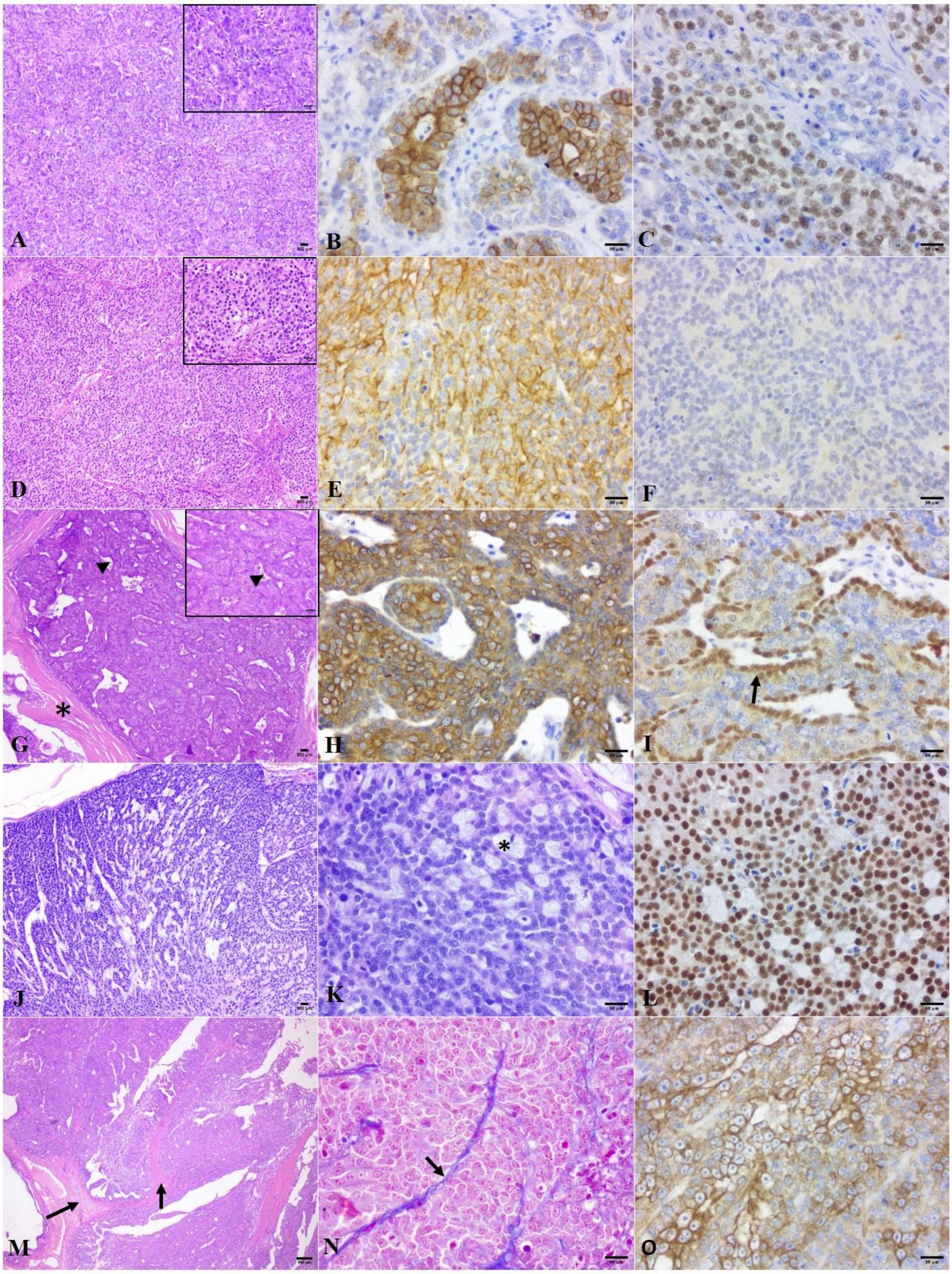

Figure S1. Canine Mammary Neoplasms with Solid Arrangement. **Malignant Adenomyoepithelioma**: A. Neoplastic cells were organized in mantles or solid nests, with scarce tubules and stroma, HE, bar = 100 µm. B. Immunolabelling for pancytokeratin in the cytoplasm of epithelial cells, bar = 50 µm. C. Immunolabelling for p63 in the nuclei of myoepithelial cells, bar = 50 µm. **Carcinomas with Solid Pattern:** D. Neoplastic cells were organized in solid nests, HE, bar = 100 µm. E. Immunolabelling for pancytokeratin in the cytoplasm of epithelial cells, bar = 50 µm. F. No immunostaining for p63, bar = 50 µm. **Basaloid carcinoma:** G. solid nests surrounded by a layer of peripheral palisading cells (arrow head), multinodular pattern separated

by moderate fibrous stroma (*), HE, bar = 100 µm. H. Central cells immunolabelled for pancytokeratin, bar = 50 µm. I. Palisading peripheral cells stained with cytokeratin 14 (arrow), bar = 50 µm. **Malignant Myoepithelioma:** J. Elongated and rounded cells oriented in various directions, HE, bar = 100 µm. K. Cells with clear cytoplasm, round nuclei, and intercellular myxoid material (*), HE, bar = 50 µm. L. Immunolabelling for p63 in the nuclei of myoepithelial cells, bar = 50 µm. **Solid Papillary Carcinomas:** M. Papillary projections with multiple layers of epithelial cells extending from the stroma (arrow), forming a solid appearance, HE, bar = 100 µm. N. Delicate fibrous stroma (arrow) supporting the layers of epithelial cells, Gomori's trichrome, bar = 50 µm. O. Immunolabelling for pancytokeratin in the cytoplasm of epithelial cells, bar = 50 µm.

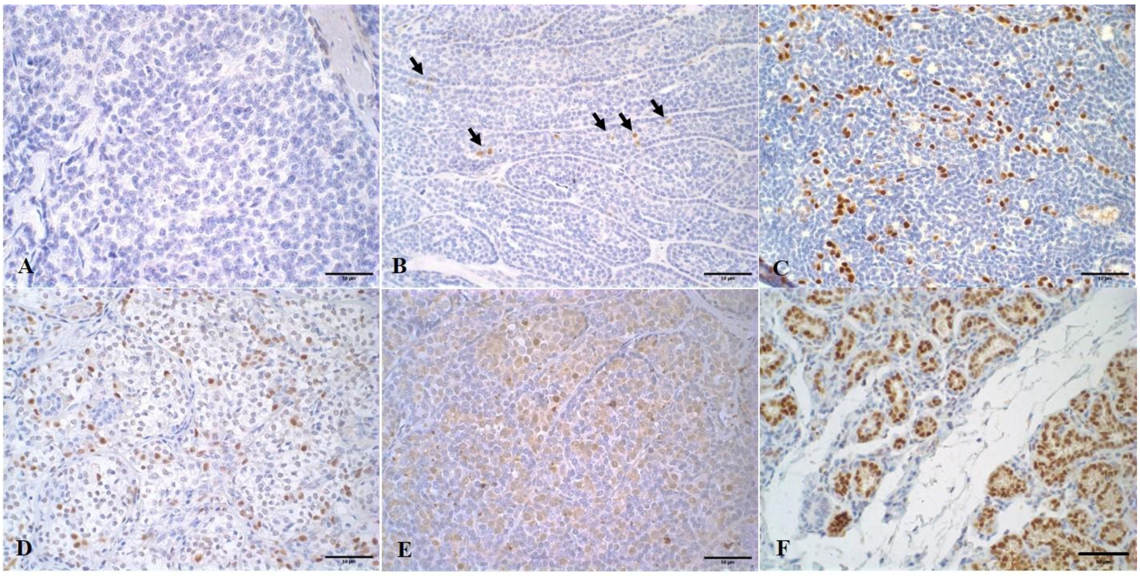

Figure S2. Immunohistochemistry for estrogen receptor in Canine Mammary Neoplasms with Solid Arrangement. A. Negative immunostaining (<1%). B. Nuclear staining in a few cells (1–25% of labeled cells) (arrow). C. 26–50% of labeled cells. D. 51–75% of labeled cells. E. More than 75% of labeled cells. F. Positive control in the mammary gland of a non-neoplastic female dog. Bar=50um.

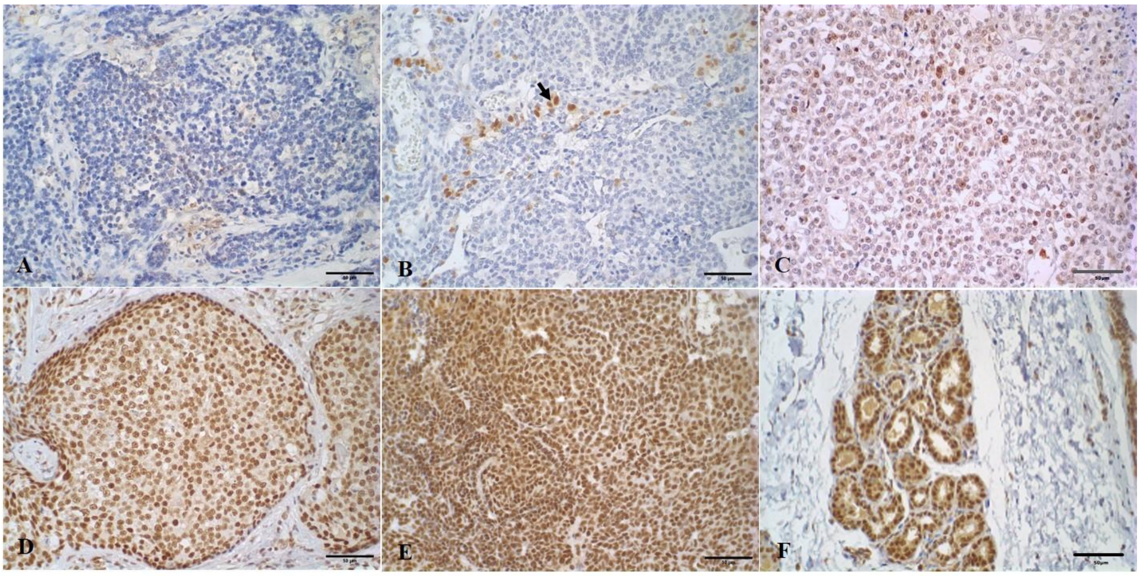

Figure S3. Immunohistochemistry for progesterone receptor in Canine Mammary Neoplasms with Solid Arrangement. A. Negative immunostaining (<1%). B. Nuclear staining in a few cells (1–25% of labeled cells) (arrow). C. 26–50% of labeled cells. D. 51–75% of labeled cells. E. More than 75% of labeled cells. F. Positive control in the mammary gland of a non-neoplastic female dog. Bar=50um.

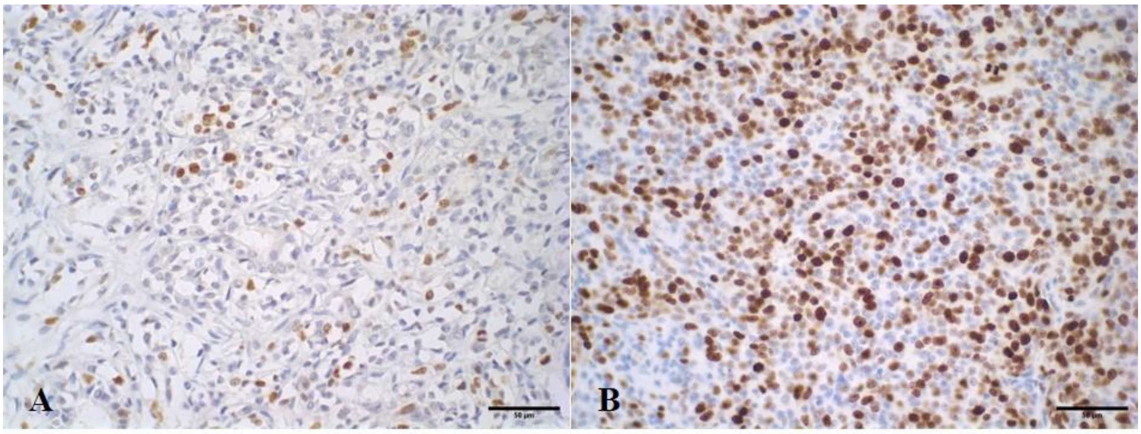

Figure S4. Immunohistochemistry for Ki-67 in Canine Mammary Neoplasms with Solid Arrangement. A. Neoplasm with a high nuclear staining count for Ki-67, showing >20% of cells with nuclear staining. B. Neoplasm with a low nuclear staining count for Ki-67, showing <20% of cells with nuclear staining. Bar=50um.

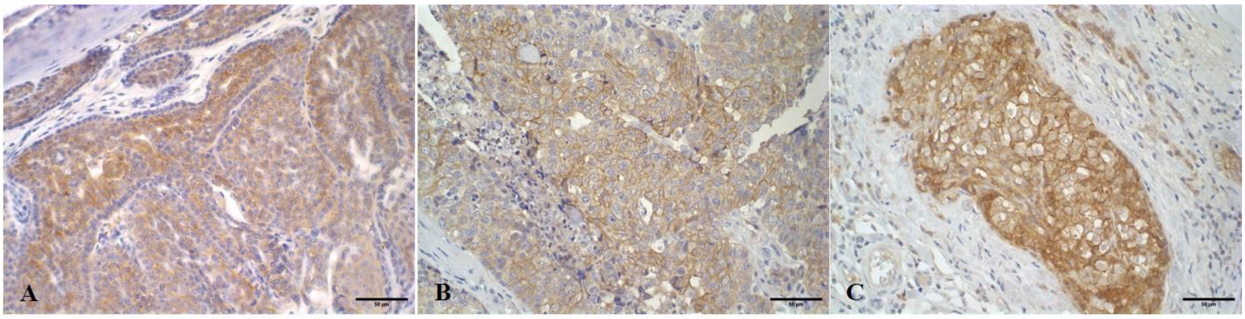

Figure S5. Immunohistochemistry for HER-2 in Canine Mammary Neoplasms with Solid Arrangement followed the guidelines of membrane marking score (Wolff, et al, 2013). A. Neoplasm with score 1. B. Neoplasm with score 2. C. Neoplasm with score 3. Bar=50um.

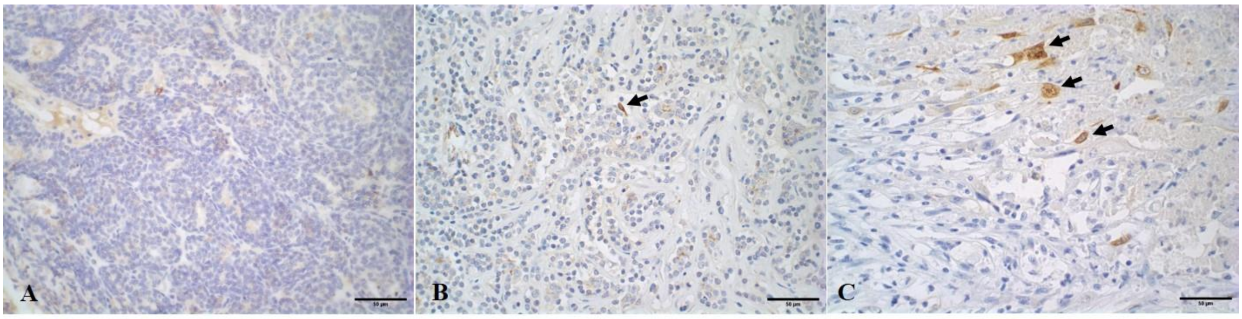

Figure S6. Immunohistochemistry for COX-2 in Canine Mammary Neoplasms with Solid Arrangement, based on a semi-quantitative evaluation of labeled tumor cells in the cytoplasm (Lavalle et al., 2009). A. Neoplasm without immunostaining for COX-2. B. Neoplasm with a score of 1 (less than 10% of cells labeled; weak intensity) (arrow). C. Neoplasm with a score of 3 (less than 10% of cells labeled; strong intensity) (arrow). Bar = 50 µm.
